# Efficient removal of toxic azo dyes from contaminated water by adsorption on the GO surface

**DOI:** 10.1371/journal.pone.0299364

**Published:** 2024-03-29

**Authors:** Haris bin Tanveer, Fouzia Perveen, Sumaiya Azam, Nasima Arshad, Hummera Rafique, Ahmad Irfan, Zoniya Arshad, Salman u Zaman, Sher Qadar

**Affiliations:** 1 School of Interdisciplinary Engineering & Sciences (SINES), National University of Sciences & Technology (NUST), Islamabad, Pakistan; 2 Department of Chemistry, Allama Iqbal Open University, Islamabad, Pakistan; 3 Department of Chemistry, University of Gujrat, Gurjat, Pakistan; 4 Department of Chemistry, College of Science, King Khalid University, Abha, Saudi Arabia; Adama Science and Technology University, ETHIOPIA

## Abstract

The purpose of this study is to examine the possibility of GO to be used as an adsorbent for five novel potentially hazardous azo-dyes for their removal from aqueous solution. Adsorption characteristics of GO for azo-dyes removal were investigated by means of experimental and computational DFT as well as Monte Carlo approaches. Experimental studies include the effect of adsorbent dose, contact time, and initial concentration, while computational investigation involves DFT and Monte Carlo (MC) simulations. Through DFT studies geometric, electronic, and thermodynamic parameters were explored and possible mechanism of interactions and adsorption energies by predicted through MC by searching lowest possible adsorption complexes. Experimental data were evaluated by Langmuir models in order to describe the equilibrium isotherms. Equilibrium data fitted well to the Langmuir model. Thermodynamic parameters i.e., free energy change, enthalpy change, and entropy change revealed that the removal of azo-dyes by adsorption on the surface of GO molecular sieves was spontaneous. Nature of the process was found to be physiosorption involving non-covalent interaction. The study unveiled that GO can be used as an efficient adsorbent material for the adsorption of azo-dyes from aqueous solution.

## 1. Introduction

Now a days over 10,000 dyes are being used in various industries including leather, plastics, rubber, cosmetic, textile, paper, food and pharmaceutical industries [1]. The release of these dyes into water is responsible for reduced photosynthetic activity due to interference with transmission of sunlight [2]. For the purpose of making color long-lasting, these dyes are designed to resist decomposition with time, exposure to sunlight, water and soap. Due to their complex structure, dyes cannot be easily removed from water by conventional wastewater treatment processes [3].

Azo dyes are organic compounds having aryl functional group. Wastewater released from factories has high percentage of Azo dyes dissolved in it which not only damage plant growth but causes metabolic stress and neurosensory damage in fish [4]. Azo dyes can come in contact with humans through a number of ways. Approx.4-5% of azo dyes decompose to form aromatic amines, which are potentially cancerous and have therefore been regulated. Specifically, they have been seen to cause bladder and liver cancers [5]. Adsorption is a robust, well studied, widely employed and promising water treatment method which is considered to be one of the most simple and economical to remove the dyes from sewages [6]. The adsorption attempts have been made to find alternative low-cost adsorbents [7].

In the last decade, nanocarbon based adsorbents have attained much attention in water treatment. These adsorbents are synthesized from low-cost materials and are proven to be highly efficient than other adsorbents. One widely used and known adsorbent material is activated carbon. Carbon nanotubes (CNT), characterized by hollow tubular structure with high surface area, have also been studied for the adsorption of a large number of different organic compounds from water [8]. Now a days, graphene-based materials such as graphene oxide (GO) and reduced graphene oxide (rGO) have been widely investigated for adsorption application [9–13]. Graphene based materials have high surface area compared to CNT and consist of sp2 and sp3 hybridized hexagonal honeycomb carbon network with abundant functional sites which are ideal for a proficient adsorbent material [14]. In GO, additionally surface defects such as vacancies and sp3 bonded atoms are most common [15]. The presence of these functional groups or defects allows GO to easily interact with organic and inorganic compounds by covalent or non-covalent interactions [16]. Moreover, GO has high dispersion ability in the aqueous solutions compared to graphite due to increased interplanar distance or weakened interplanar π–π interactions, the GO may regain sufficient conjugated structure of graphene which are vital for any adsorbents of organic dyes, particularly for aromatic structures [17, 18]. In one of our previous work, we presented as simple and facile method for the reduction of silver salt in the presence of GO using Azo dyes including EBT and MO for developing composites. Wojciech Konicki *at al*, performed experimental studies for the removal of industrial acid Orange 8 (AO8) and Direct Red 23 (DR23) dyes by adsorption onto graphene oxide [19].

The aim of this work is to study the removal of five novel dyes which are released into water as a result of chemical laboratory synthesis. These dyes are classified as azo-dyes, and a majority of azodyes exhibit toxicity, carcinogenicity, and mutagenicity, leading to adverse effects such as allergies, dermatitis, skin irritation, cancer, and mutations in both humans and animals [20, 21].

We present here the removal of five novel azo dyes through graphene oxide as the adsorbent using both computational approaches and experimental UV-Vis spectroscopy. Computational method play a crucial role in studying adsorption process for water purification, offering several advantages that enhance our understanding of the process. In the realm of water purification through adsorption, these methods provide detailed insights into the molecular interactions between adsorbent materials and contaminants at the atomic level. For understanding the specific adsorption mechanisms, binding energies, and thermodynamics at the molecular scale, DFT and Monte Carlo simulations were performed to determine the adsorption energy of the dyes and efficiency GO for water purification [22].

Experimentally, to comprehend the adsorption mechanism of azo-dyes onto graphene oxide, a comprehensive analysis of the equilibrium, kinetic, and thermodynamic data for adsorption was conducted by using UV-Vis spectroscopic investigation.

The kinetics and thermodynamics of the adsorption were also measured by using both computational and experimental approaches. Nature of adsorption was explored to ensure recycling of graphene oxide surface. To the best of our knowledge, computational prediction and then experimental validation for the adsorptive removal of these novel dyes on GO surface through diverse computational and experimental approaches is reported for the first time.

## 2. Materials and methods

### 2.1 Synthesis of graphene oxide

In modified Hummer’s method conc. HNO_3_ and KMnO_4_ were utilized to synthesize GO from graphite powder. It is a safer method with the reduced preparation time of GO. For the synthesis of expanded graphite, 30 ml of conc.H_2_SO_4_ was added into 250mlbeaker and placed it on a hot plate for stirring, conc. HNO_3_ was added dropwise, 15g of graphite were added, beaker was covered with a lid and solution was left soaked for three days. After 3 days entire mixture was transferred into 200mldeionized water for intercalation of graphite to get expanded graphite. The mixture was centrifuged for 30 mins, two layers were generated, upper layer was decanted while lower layer of black precipitate was warmed in oven at 70 degrees for 3–4 hrs. After heating the intercalation of graphite was obtained which is highly hydrophilic and effectively be oxidized.

Oxidation of expanded graphite was performed by the addition of KMnO_4_ in the presence of conc.H_2_SO_4_ with continuous stirring. During this process 4g of expanded graphite was added in three intervals. The solution was placed in an ice bath and proceeded with stirring. This solution was then placed in an ice bath for controlling the sudden elevation in temperature. After 1hour 375 ml of deionized water was added and placed it in the water bath at 90°C followed by addition of H_2_O_2_ for the removal of remnants of manganese. After addition of hydrogen peroxide, mixture was centrifuged at 6000rpm and washed with deionized water.

To this mixture 1ml of 1M solution HCl was added and repeatedly at deionized water to remove permanganate and manganese dioxide salts. This layer-by-layer washing is known as chemical exfoliation. Mixture was taken and placed on hot plate with stirring for 1hr. Now precipitates were dried at 60–70°C as dried precipitates of graphene oxide [23].

### 2.2 General method for synthesis of azo dyes

Amine-I (4,4’-methylene dianiline) was weighed in grams on weighing balance. The weighed amine were dissolved into 20ml of dry methanol in a round bottom flask. The flask was placed in the ice bath on stirring plate and by using the thermometer the temperature was maintained at 0–5°C. Then added the double moles of HCl in amount of 2ml on stirring and kept temperature at the range 0–5°C. NaNO_2_ solution was prepared by weighed amount into the distilled water and was added drop wise into the reaction flask by using the dropper at the set temperature of 0–5°C. The contents of the reaction flask were stirred for 1hr till pink color appeared. Aldehyde (weighed amount) were added by making the solution of it into the required amount of Methanol keeping the temperature same. Put the aldehyde solution into the reaction flask drop wise at maintained temperature. Then allowed the reaction to stir for further 3hrs until color changed into orange brown shade. The reaction was monitored by TLC after completion, neutralized the solution at the same temperature by adding the sodium hydroxide (NaOH) solution drop wise. The precipitates were appeared and the reaction mixture was removed from the ice bath and filtered it. After washing precipitates were allowed the to dry. The general reaction for the synthesis of the Azo dyes from Amine-1 is given in the S1 Scheme.

**Fig 1:**
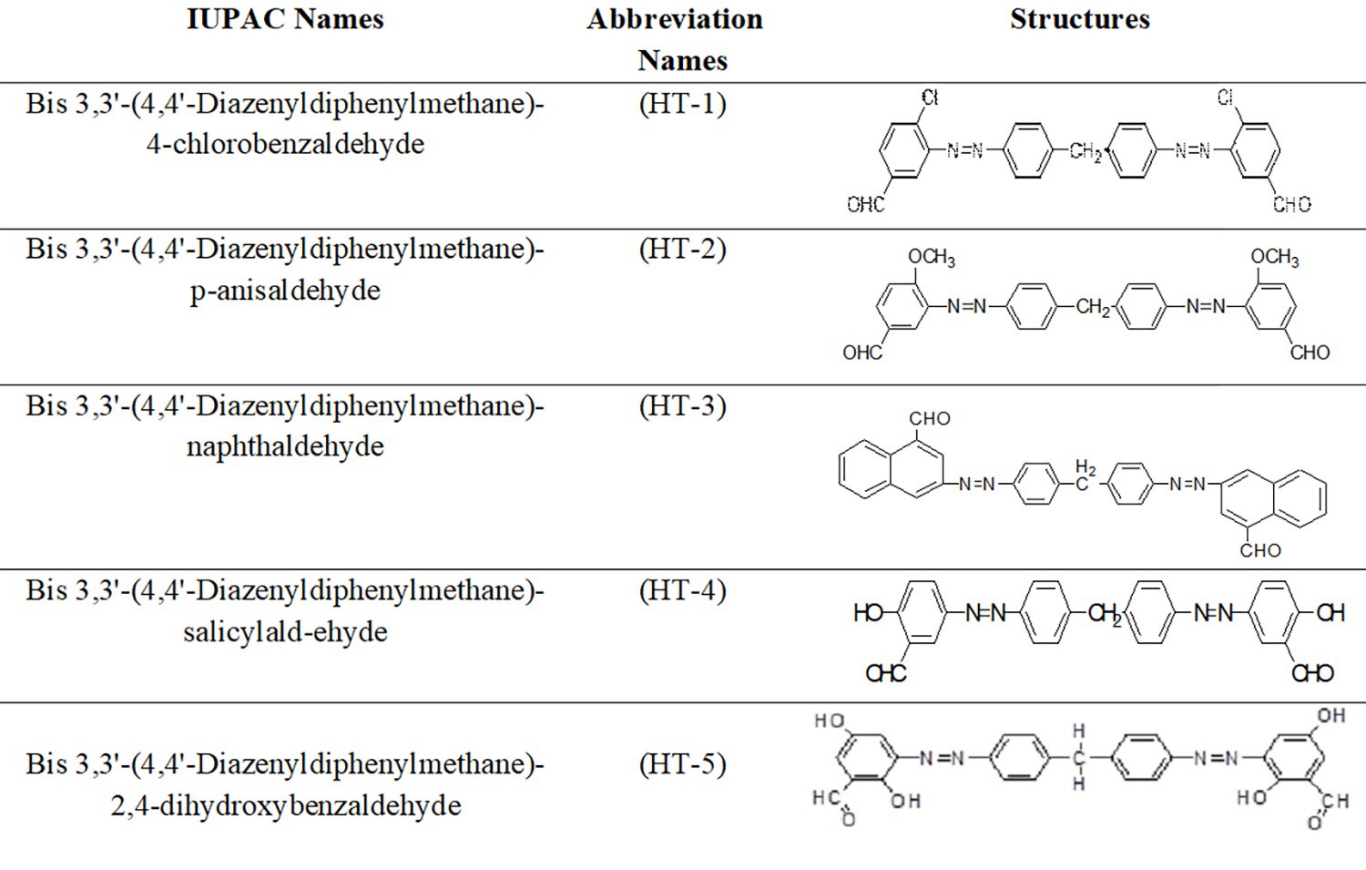
Molecular structures and IUPAC names of five novel azo dyes used as adsorbates.

In the present work following five azo dyes were synthesized by using above scheme as mentioned in Fig 1.

### 2.3 UV-vis spectroscopic procedure

Adsorption studies for azo dyes have been carried out to investigate the effect of different parameters such as adsorbate concentration, adsorbent dose, solution containing various concentrations of adsorbate (ppm) and 0.05g adsorbent was taken in 100ml (1:9; DMSO: water) mixture in conical flask and agitated at 150rpm in orbital shaker. The initial and final azo-dyes concentrations remaining in solutions were analyzed by a UV spectrophotometer (Shimadzu-1800), monitoring the absorbance changes at a wavelength ranges 200-800nm. The equilibrium adsorption capacity was calculated from the relationship below [24].

$$q_{e}\left(\frac{mg}{g}\right)=\frac{C_{i}-C_{e}}{C_{e}}x\frac{V}{m}$$ (1)

Where *C*_*i*_ and *C*_*e*_ are the initial and equilibrium concentrations of aniline in ppm, *q* is the adsorption capacity in mg/g, V is the volume of azo-dyes solution in L, and m is the adsorbent mass in g.

### 2.4 Quantum chemical DFT calculation

Quantum chemical density functional theory (DFT) studies were performed by using SCM-ADF modeling suite 2018.105 for the adsorption kinetic studies of five novel azo dyes on the surface of GO molecular sieves. X-ray crystallographic structure of GO was obtained, modelled, and imported to ADF graphical interface as adsorbent. Optimized structures of five azodyes were then adsorbed on the surface of GO to study electronic, kinetic, and thermodynamic effects of heterogeneous adsorption. All calculations were carried out using SCM-ADF [25] employing the generalized gradient approximation (GGA) due to Perdew-Burke Ernzerhof (PBE) exchange correlation [26]. The basis sets representing the electron density consist of both Hermane Skillman numerical atomic orbitals (NAOs) and Slater-type orbitals (STOs) with a frozen core. Scalar relativistic corrections were included through the zeroth order regular approximation [27]. A three-dimensional translational symmetry was implemented for the single layer of GO. In our computation, overall energy convergence was well within 0.1eV, at LDA-GGA level of theory. Adsorption energy was calculated to determine efficiency of azo dyes binding on the GO surface. Thermodynamic parameters calculated at temperature provided insight into stability of adsorbent in azo dye removal process.

### 2.5 Monte Carlo simulations

Molecular simulations were implemented using Material Studio for exploring adsorption mechanisms and interactions between azo dyes and graphene oxide. Firstly, azo dyes and graphene oxide were optimized using the Forcite module in Material Studio software. The energy setting was UNIVERSAL forcefield with fine quality. The obtained optimized structures then were used for studying adsorption mechanisms and to identify the lowest energy adsorption location of dyes on GO using the Adsorption Locator tool in Material Studio. This module works by performing Monte Carlo searches of configurational structures of the Dyes-GO complex as the T is gradually decreased. The Monte Carlo simulations were performed for 100 annealing cycles, with same energy setting as used in geometry optimization.

## 3. Result and discussion

### 3.1 Characterizations of graphene oxide (GO)

GO characterization was done by FT-IR, XRD, TGA, SEM, and UV-spectroscopy and the related spectrum and graphs are provided in S1A–S1E Fig. In FT-IR of GO, the peak at 3200–3400 cm^-1^ indicated the O-H stretching groups, the peak at 1740.85 cm^-1^ recognized for C = O stretch, and a band at 1045.79 cm^-1^ indicated (epoxy) groups. In XRD, GO showed intensive sharp peak centered at 2θ = 12.09°, corresponding to the (002) inter planar spacing of 0.74 nm. In TGA of GO, two step decomposition was observed. The first step (100–245.6°C) is due to oxygen containing functionalities i.e.–COOH, -OH, -C = O, while in the second step (245.6–707.5°C) degradation in graphitic backbone has occurred. A sheet like structure in SEM micrograph indicated the exfoliation phenomena of GO. UV- spectrum of GO showed peak at λ_max_ at 235 nm which could be attributed to the π-π electronic transition of aromatic groups, while a shoulder peak at 300 nm depicted the presence of carbonyl groups (C = O).

The low cost, recyclability, and stability are the key factors to ascribe the efficiency of a catalyst or adsorbent [28–30]. A recycle usage experiment was performed and dispersions were filtered, cleaned with deionized water, and dried. To ascertain the stability of recycled GO, its FTIR was done, and the spectrum showed no significant change, hence assured its stability to be re-used as an absorbent for fresh dye solution. The FTIR of used adsorbent (GO) is provided as S1F Fig.

### 3.2 Characterization of synthetic azo dyes

All the synthesized Azo compounds were characterized by IR-spectroscopy, the overall range for different functionalities in IR spectra were interpreted. The characteristic peaks for aromatic C = C in IR were observed in the region from 1575–1610 cm^-1^. The fundamental signals for Azo compounds -N = N were observed in the range from 1436–1478 cm^-1^. The carbonyl C = O peaks were appeared in the region 1653–1684 cm^-1^ and the peaks for C-N functionality is observed in the range 1506–1516 cm^-1^.

The structural confirmation was also carried out by 1H and 13C-NMR analysis. In the 1H-NMR data of compounds (HT-1-5), the appearance of characteristic signals at 9.86–10.06 ppm for (aldehydic C = O) and 3.84–3.89 ppm for (Ar-CH2) confirmed the synthesis of compounds of our interest. In the 13C-NMR data the characteristic signals were observed at 198.4–199.6 ppm for (aldehydic C = O) and 44.3–44.9 ppm for (Ar-CH2). Elemental analysis data and the appearance of characteristic signals in the FR-IR and NMR spectra confirmed the synthesis of our desired Azo compounds. Elemental analysis also indicate the successful synthesis of our target Azo compounds. Representative IR and NMR spectra for HT-2 are provided in S2 Fig, whereas the data is provided as below.

#### Bis 3,3’-(4,4’-Diazenyldiphenylmethane)-4-chlorobenzaldehyde (HT-1)

Color: Dark Red, Yield: 76%, melting point/decompose: 288–290°C.; IR (KBr, υ_max_); 1589(C = C), 1436 (N = N), 1659 (C = O), 1514(C-N) cm^_1^.; ^1^H NMR (300 MHz, CDCl_3_, δ ppm): 9.93 (2H, s, -CHO), 8.61 (4H, d, *J* = 7.6 Hz, Ar-H-1’), 8.17 (2H, d, *J* = 2.4 Hz, H-2), 8.07 (2H, dd, *J* = 7.4,2.4 Hz, H-6), 7.86 (2H, dd, *J* = 7.4 Hz, H-5), 7.54 (4H, d, *J* = 7.6 Hz, Ar-H-2’), 3.88 (-CH_2_).; ^13^C NMR (75 MHz, CDCl_3,_ δ ppm): 198.4 (aldehydic C = O), 154.2 (C-3,3), 152.5 (C-3’3’), 145.7 (C-4’,4’), 136.5 (C-4,4), 133.4 (C-1,1), 131.3 (C-6,6), 129.1 (C-5,5), 128.2 (C-2,2), 125.4 (2xC-1’,1’), 124.2 (2xC-2’,2’), 44.3 (-CH_2_).; Anal. Calcd for C_27_H_18_N_4_O_2_Cl_2_ (500): C, 64.68; H, 3.62; N, 11.17; Found: C, 64.64; H, 3.57; N, 11.19%.

#### Bis 3,3’-(4,4’-Diazenyldiphenylmethane)-*p*-anisaldehyde (HT-2)

Color: Brown, Yield: 75%, melting point/decompose: 190–192°C.; IR (KBr, υ_max_); 1597 (C = C), 1449 (N = N), 1670 (C = O), 1507 (C-N) cm^_1^.; ^1^H NMR (300 MHz, CDCl_3_, δ ppm): 9.88 (2H, s, -CHO), 8.57 (4H, d, *J* = 7.6 Hz, Ar-H-1’), 8.21 (2H, d, *J* = 2.4 Hz, H-2), 8.12 (2H, dd, *J* = 7.4,2.4 Hz, H-6), 7.78 (2H, dd, *J* = 7.4 Hz, H-5), 7.64 (4H, d, *J* = 7.6 Hz, Ar-H-2’), 3.92 (-OCH_3_), 3.87 (-CH_2_).; ^13^C NMR (75 MHz, CDCl_3,_ δ ppm): 198.2 (aldehydic C = O), 154.7 (C-3,3), 153.4 (C-3’3’), 146.3 (C-4’,4’), 137.5 (C-4,4), 134.2 (C-1,1), 132.8 (C-6,6), 131.3 (C-5,5), 130.5 (C-2,2), 126.3 (2xC-1’,1’), 124.7 (2xC-2’,2’), 56.2 (-OCH_3_), 44.6 (-CH_2_).; Anal. Calcd for C_29_H_24_N_4_O_4_ (492): C, 70.72; H, 4.91; N, 11.38; Found: C, 70.74; H, 4.95; N, 11.36%.

#### Bis 3,3’-(4,4’-Diazenyldiphenylmethane)naphthaldehyde (HT-3)

Color: Orange, Yield: 62%, melting point/decompose: 180–182°C.; IR (KBr, υ_max_, cm^-1^); 1575 (C = C), 1457 (N = N), 1684 (C = O), 1507 (C-N) cm^_1^.; ^1^H NMR (300 MHz, CDCl_3_, δ ppm): 9.91 (2H, s, -CHO), 8.87 (4H, d, *J* = 7.6 Hz, Ar-H-1’), 8.76–7.68 (12H, m, Ar), 7.61 (4H, d, *J* = 7.6 Hz, Ar-H-2’), 3.86 (-CH_2_).; ^13^C NMR (75 MHz, CDCl_3,_ δ ppm): 198.8 (aldehydic C = O), 152.4 (C-3’3’), 148.7 (C-3,3), 145.4 (C-4’,4’), 138.4 (C-4,4), 137.5 (C-1,1), 134.6 (C-10,10), 133.2 (C-9,9), 132.5 (C-2,2), 131.4 (C-5,5), 130.5 (C-6,6), 129.7 (C-7,7), 128.5 (C-8,8), 127.4 (2xC-1’,1’), 125.6 (2xC-2’,2’), 44.5 (-CH_2_).; Anal. Calcd for C_35_H_24_N_4_O_2_ (532): C, 78.93; H, 4.54; N, 10.52; Found: C, 78.97; H, 4.55; N, 10.53%.

#### Bis 3,3’-(4,4’-Diazenyldiphenylmethane)salicylaldehyde (HT-4)

Color: Brown, Yield: 68%, melting point/decompose: 198–200°C.; IR (KBr, υ_max_); 1598 (C = C), 1478 (N = N), 1653 (C = O), 1506 (C-N) cm^_1^.; ^1^H NMR (300 MHz, CDCl_3_, δ ppm): 10.03 (2H, s, -CHO), 8.66 (4H, d, *J* = 7.6 Hz, Ar-H-1’), 8.26 (2H, dd, *J* = 7.4,2.3 Hz, H-4), 7.96 (2H, dd, *J* = 7.4,2.4 Hz, H-6), 7.81 (2H, dd, *J* = 7.4,7.4 Hz, H-5), 7.67 (4H, d, *J* = 7.6 Hz, Ar-H-2’), 5.46 (-OH), 3.86 (-CH_2_).; ^13^C NMR (75 MHz, CDCl_3,_ δ ppm): 199.2 (aldehydic C = O), 158.5 (C-2,2), 154.8 (C-3,3), 153.6 (C-3’3’), 146.9 (C-4’,4’), 138.3 (C-4,4), 136.6 (C-1,1), 134.1 (C-6,6), 132.7 (C-5,5), 126.8 (2xC-1’,1’), 125.6 (2xC-2’,2’), 44.7 (-CH_2_).; Anal. Calcd for C_27_H_20_N_4_O_4_ (464): C, 69.82; H, 4.34; N, 12.06; Found: C, 69.88; H, 4.45; N, 12.03%.

#### Bis 3,3’-(4,4’-Diazenyldiphenylmethane)-2,4-dihydroxybenzaldehyde (HT-5)

Color: Yellow, Yield: 68%, melting point/decompose: 178–180°C.; IR (KBr, υ_max_); 1610 (C = C), 1445 (N = N), 1683 (C = O), 1516 (C-N) cm^_1^.; ^1^H NMR (300 MHz, CDCl_3_, δ ppm): 10.06 (2H, s, -CHO), 8.83 (4H, d, *J* = 7.4 Hz, Ar-H-1’), 7.96 (2H, d, *J* = 2.4 Hz, H-4), 7.84 (2H, d, *J* = 2.4 Hz, H-6), 7.72 (4H, d, *J* = 7.4 Hz, Ar-H-2’), 5.48, 5.54 (-OH), 3.89 (-CH_2_).; ^13^C NMR (75 MHz, CDCl_3,_ δ ppm): 199.6 (aldehydic C = O), 158.6 (C-2,2), 157.4 (C-4,4), 154.8 (C-3,3), 153.8 (C-3’3’), 137.3 (C-1,1), 134.5 (C-6,6), 133.7 (C-5,5), 127.4 (2xC-1’,1’), 126.5 (2xC-2’,2’), 44.9 (-CH_2_).; Anal. Calcd for C_27_H_20_N_4_O_6_ (496): C, 65.32; H, 4.06; N, 11.29; Found: C, 65.37; H, 4.05; N, 11.33%.

#### 3.3 Effect of adsorbent dosage on the adsorption capacity

**Fig 2:**
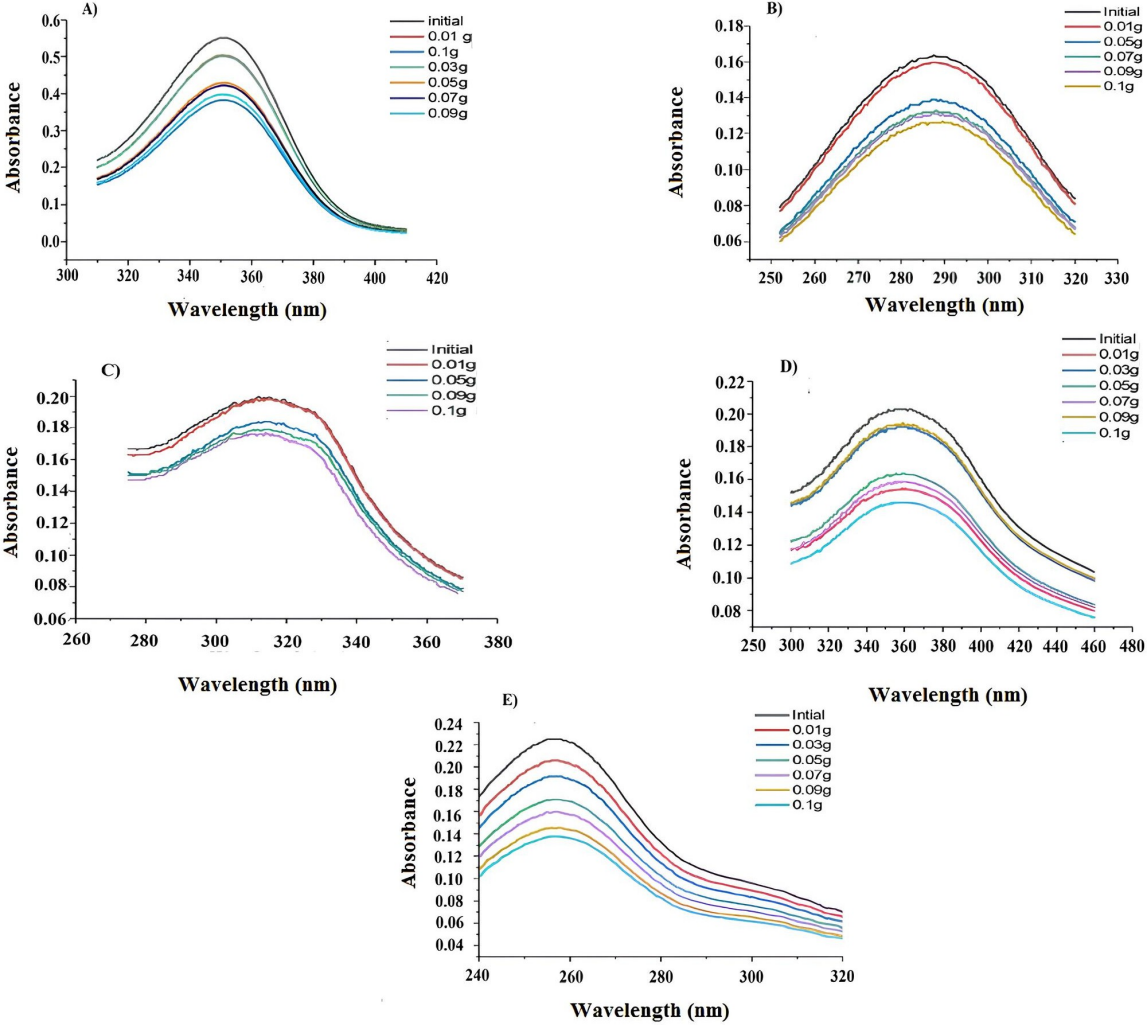
UV-Vis Spectral representation of the effect of Graphene Oxide dosage at a fixed concentration of azo-dyes A) (HT 1–1), B) (HT 1–2), C) (HT 1–3), D) (HT 1–4), and E) (HT 1–5) at 298K temperature.

The results of experiments to get insight into the effects of adsorbent dosage on the removal of each azo-dye removal are shown in Fig 2. UV-Vis spectroscopic representation depicted variation in absorbance and hence concentration of azo dyes upon GO addition [31]. Hypochromism in Fig 2A–2E revealed that absorbance and hence concentration of azo dyes was decreased with increase in concentration of GO. However, a prompt decrease in concentration of azo dyes was observed at GO dosages ranging between 0.01g and 0.1g reflecting significant adsorption and hence interaction of azodyes with at specific dosage of GO. Increase of GO dosage above 0.05g had meager effect on the decrease in concentration of azo dyes indicative of the fact that azo dye removal efficiency is maximum for GO concentration between 0.01 and 0.1g and further increase in its concentration has not effect on azo dye removal.

It is apparent from Fig 1 that there was no shift in λ_max_ demonstrating physiosorption nature of the process. Physisorption is favorable for recycling GO after adsorption as it is attributed to the non-covalent interactions between GO and azo-dyes, hence making feasible removal of azodyes from water.

### 3.4 Effect of contact time and initial azo-dyes concentrations on the adsorption capacity

**Fig 3:**
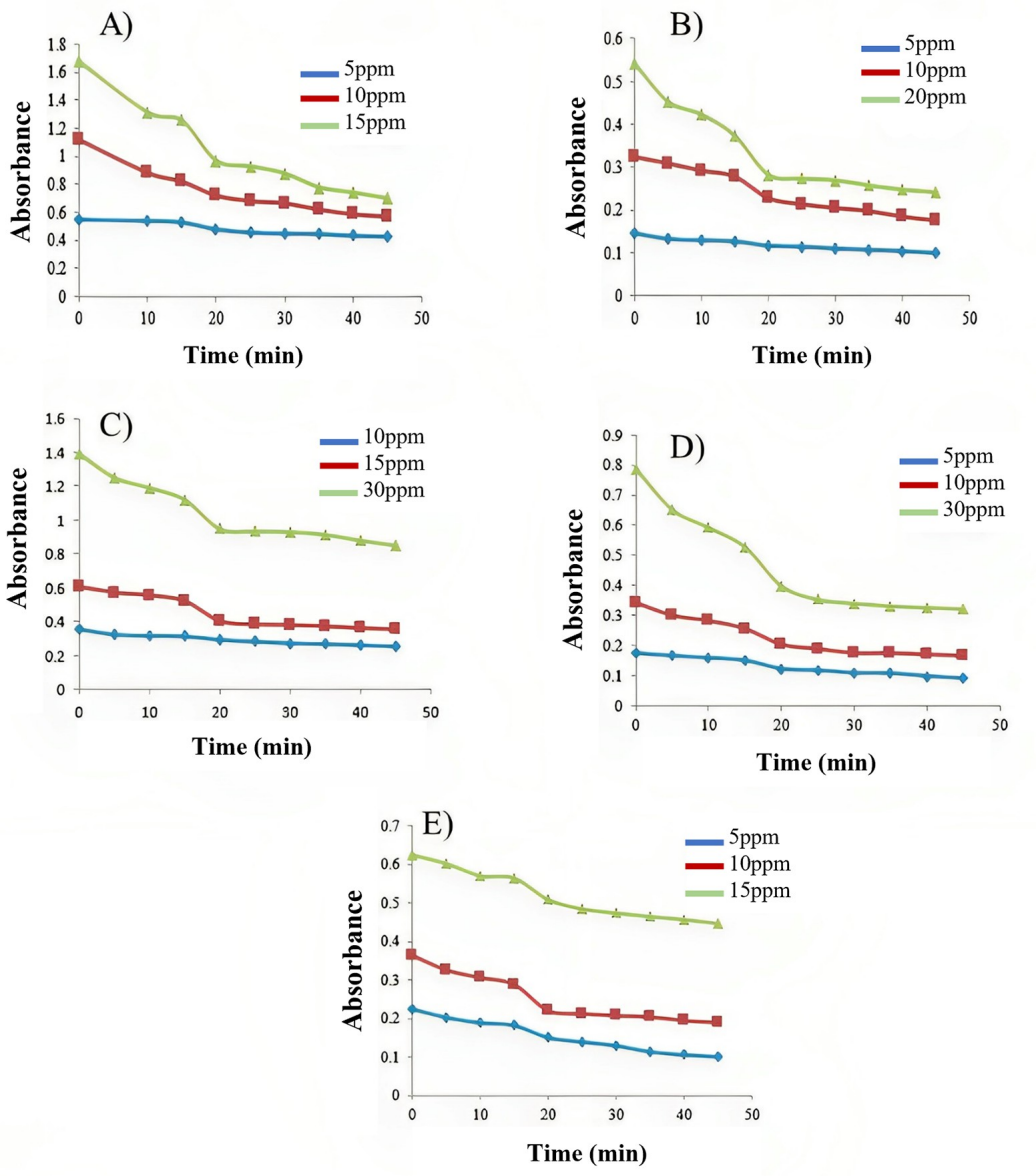
Graphical representation of Contact time study using 0.05g quantity of Graphene Oxide at various concentrations of azo-dyes A) (HT 1–1), B) (HT 1–2), C) (HT 1–3), D) (HT 1–4), and E) (HT 1–5) at 298K temperature by means of UV-Vis spectroscopy.

The effect of the initial concentration of azo-dyes on their removal rate at 0.05g GO dosage is represented in Fig 3. It is evident that the adsorption at different concentrations is fast at the initial stages and gently reduces with the progress of adsorption until the equilibrium is established. This indicated that saturation of GO surface reduced adsorption rate of azodyes and is increased after surface refreshing of GO which can be feasibly achieved due to physiosorption. The amount of azo dyes adsorbed at equilibrium (q_e_) was increased as the initial concentration of azodyes was enhanced at equilibrium. Therefore, a higher initial concentration of azo dyes improve the sorption process. The azo-dyes removal efficiency remained constant as the azo-dyes concentration was increased after equilibrium. Fig 3 also indicated that the adsorption process gradually increased with the increase of contact time and it stayed constant after adsorption equilibrium was acquired at about 20mins which is equilibrium time.

### 3.5 Adsorption isotherms

Equilibrium data, formally recognized as adsorption isotherms, have significant importance in the basic principles of adsorption processes, and are critical in optimizing the use of adsorbents [32]. To optimize the design of an adsorption system for removal of azodyes from solutions, it is essential to establish the most appropriate correlation for the equilibrium curves. For the examination of the controlling mechanisms of adsorption process, Langmuir adsorption isotherm model is used for the adsorption of molecules on solid surface forming a monolayder on the surface [33]. In this work, Langmuir adsorption isotherm was used for each azo-dye molecule to be adsorbed on GO surface and for finding K_L_ and this further utilized for finding the ΔG for spontaneity of reaction.

**Fig 4:**
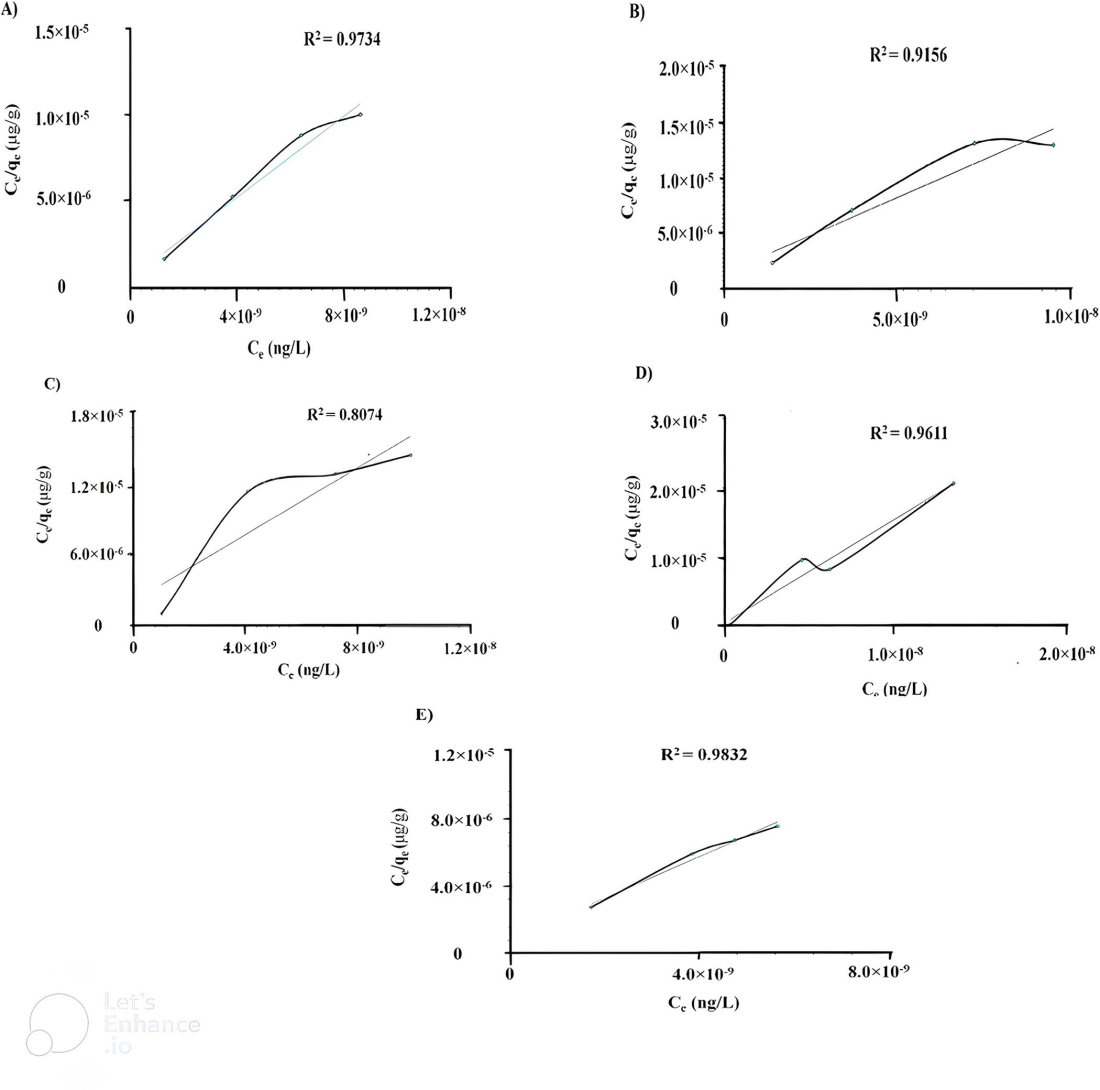
Graphical representation of Langmuir adsorption isotherm of adsorbed azo-dyes A) (HT 1–1), B) (HT 1–2), C) (HT 1–3), D) (HT 1–4), and E) (HT 1–5) on GO at 298K temperature using of UV-Vis spectroscopy.

Fig 4. depicted the graphical representation of Langmuir adsorption equation (Eq 2) for all five novel azo-dyes on the surface of GO and revealed that Bis 3,3’-(4,4’-Diazenyldiphenylmethane)-salicylaldehyde (HT-4) has highest Langmuir adsorption constant and hence strongest adsorption on the surface of GO because of intermolecular interaction of its -OH and -CHO groups coupler with graphene oxide–COC- group.

$$\frac{C_{e}}{q_{e}}=\frac{1}{K_{L}q_{m}}+\frac{C_{e}}{q_{m}}$$ (2)

Where, C_e_ = equilibrium concentration (mg/L), q_e_ = amount adsorbed per unit wight of adsorbent, K_L_ = Langmuir adsorption constant, q_m_ = maximum adsorbed amount.

### 3.6 Adsorption kinetics

Various models can be employed to initiate the understanding of the solute adsorption process onto an adsorbent. Research efforts are undertaken to develop a rapid and efficient model for the design, focusing on the adsorption rate. For monitoring the process of adsorption, kinetics models were used to the explain experimental data.

#### 3.6.1 Pseudo-first-order kinetic model

The rate constant for the adsorption of dyes is represented by the pseudo-first-order kinetic model according to the Eq (3) as proposed by [34].


$$.ln(q_{e}‐q_{t})=ln(q_{e})–k_{1}t$$ (3)

**Fig 5:**
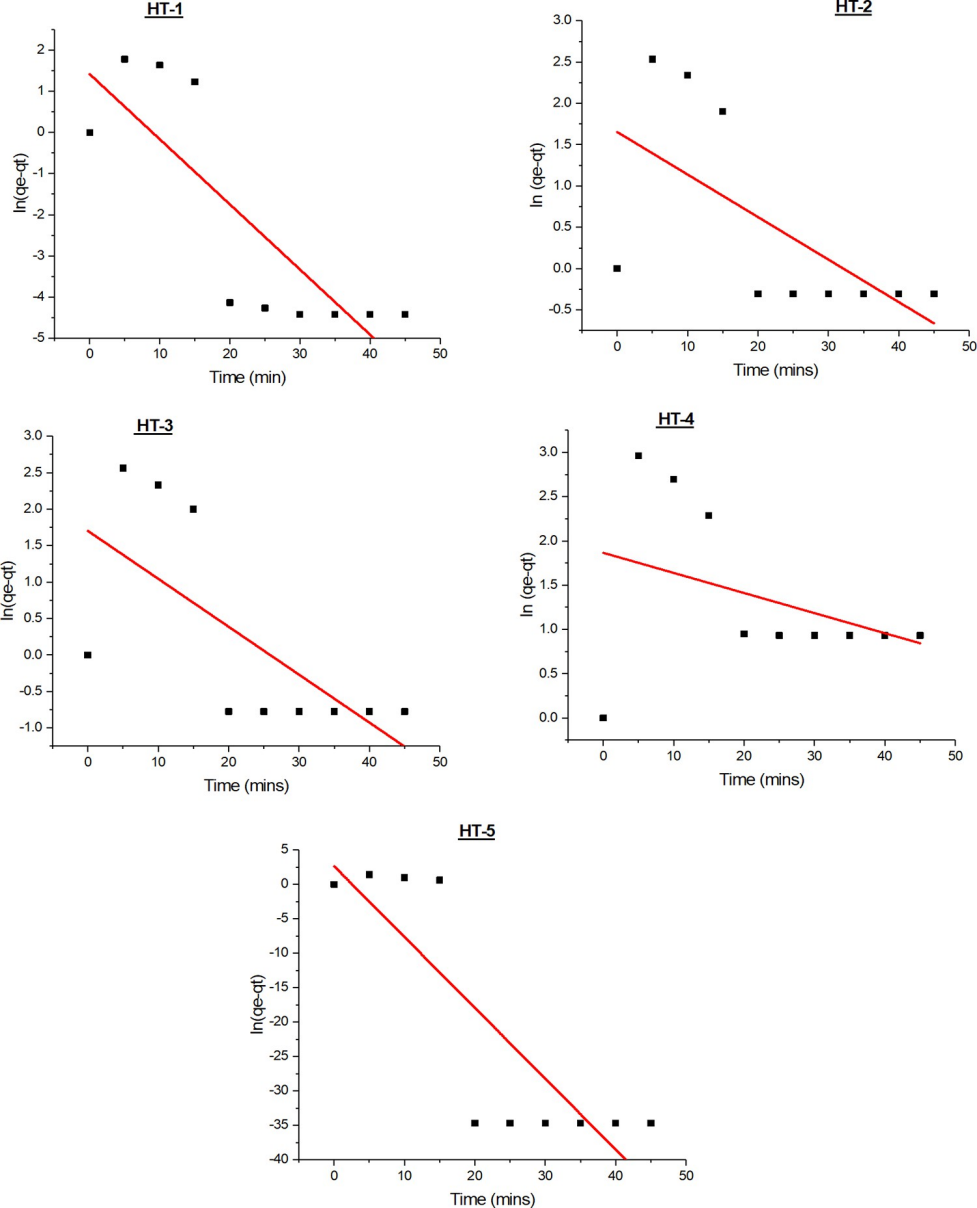
Pseudo-first-order kinetic modeling of azo-dyes adsorption on GO.

Here, q_e_ and q_t_ are the amounts of adsorbate adsorbed (mg/g) at equilibrium and at any instant of time t (min), respectively, k_1_ is the rate constant of pseudo-first-order adsorption (min^-1^.) For Pseudo first orde model, k_1_ and qe values were determined from the slope and intercept of the eq(3) as indicated in the Fig 5.

#### 3.6.2 Pseudo-second-order kinetic model

**Fig 6:**
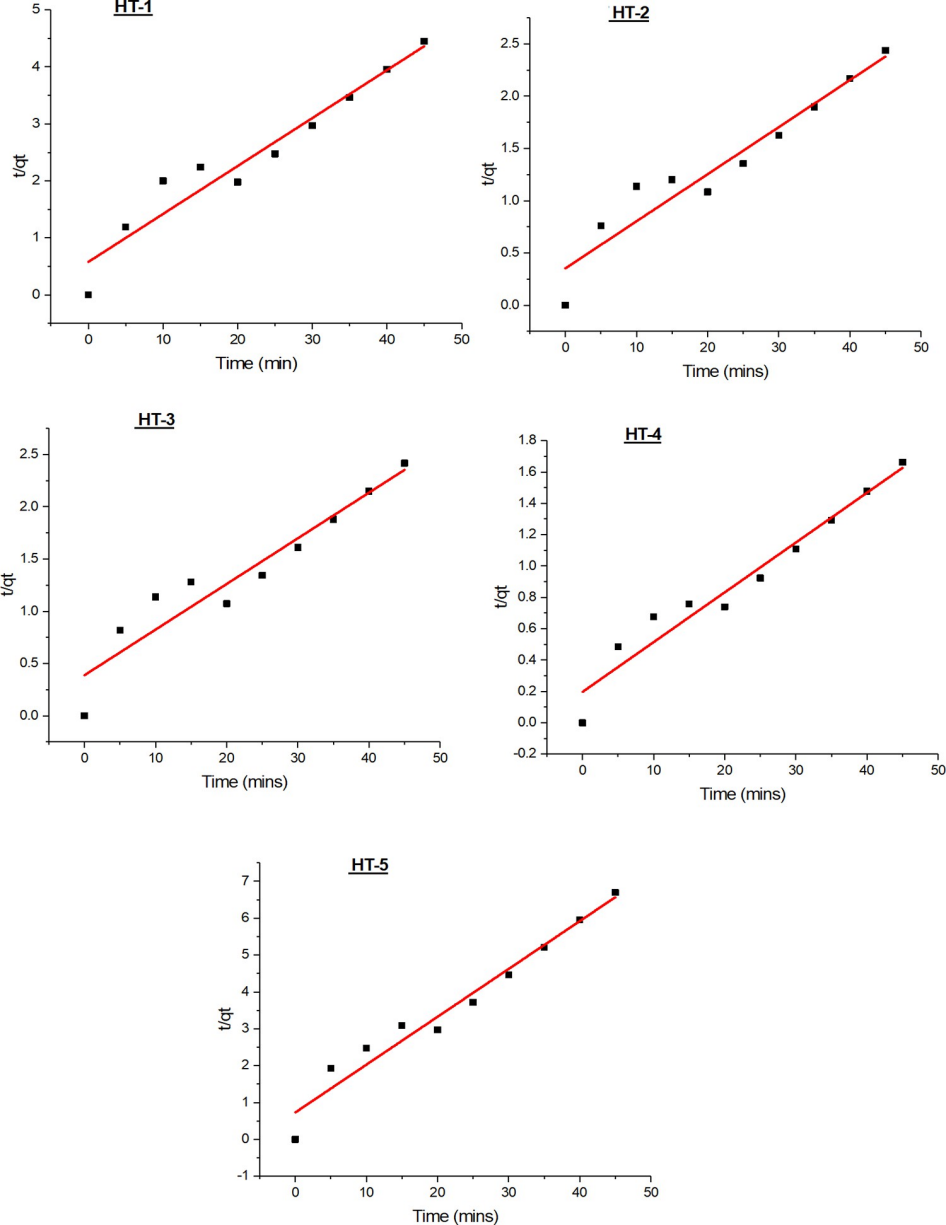
Pseudo-second-order kinetic modeling of azo-dyes adsorption on GO.

Based on equilibrium adsorption, the pseudo-second-order kinetic model, can be represented in the form as below.

$$\frac{t}{q_{t}}=\frac{1}{k_{2}\;q_{e}^{2}}+\frac{t}{q_{e}}$$

where k_2_ is the equilibrium rate constant of pseudo-second-order adsorption (g/(mg min)). A plot of t/qt vs. t (Fig 6) provides a linear relationship, rendering q_e_ and k_1_ from the slope and intercept of the plot, respectively. Experimental data used for the Pseudo-first-order and second-order kinetic models has been provided as S1 Table.

### 3.7 Computational methods and models

#### 3.7.1 Quantum chemical DFT calculation

**Fig 7:**
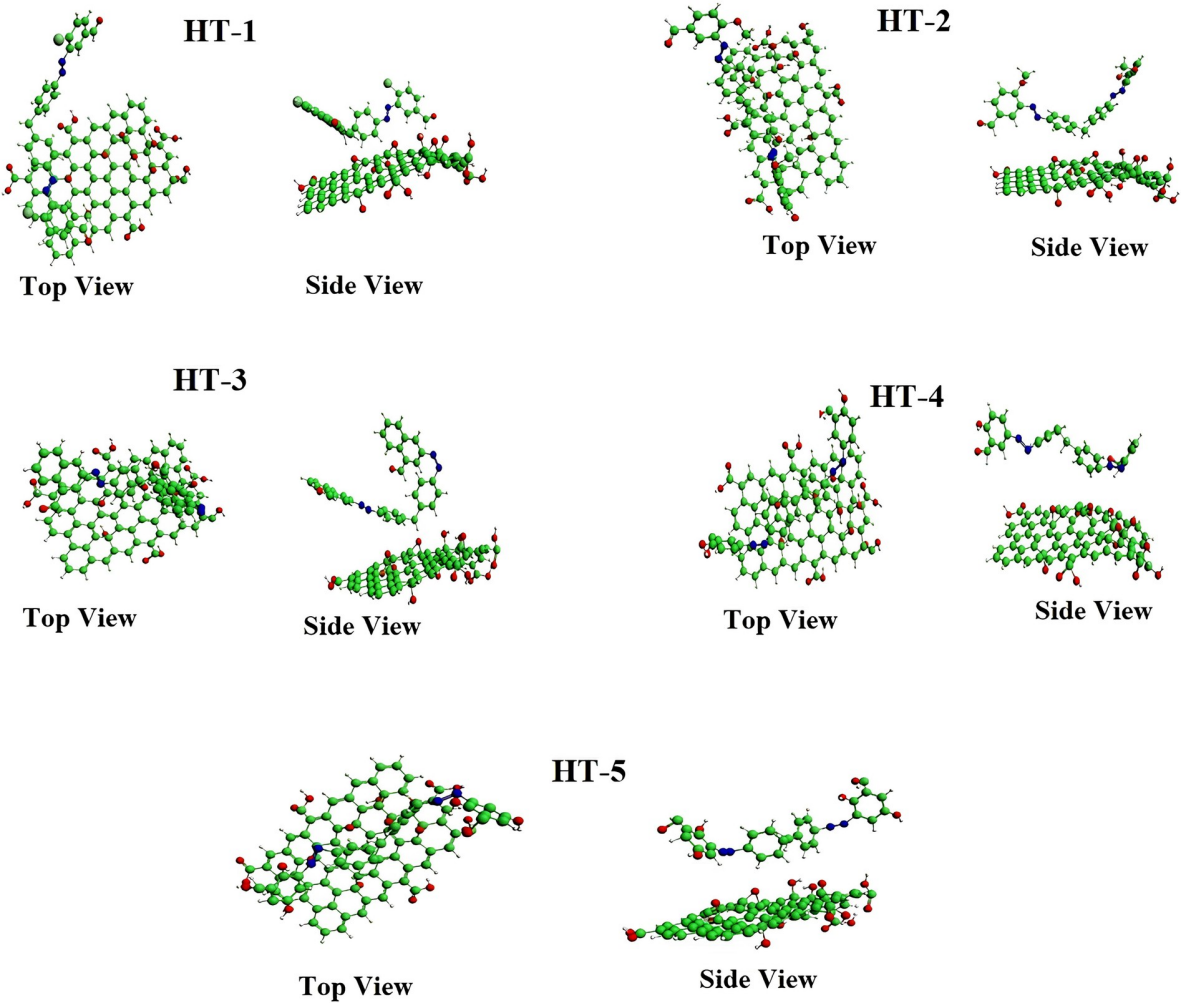
Top view and side view of optimized geometries of GO surface with adsorbed azodyes calculated through DFT studies at LDA-GGA:PBE level of theory.

Computational quantum mechanical DFT studies were performed for finding the kinetics and mode of adsorption as well as nature of adsorption (physiosorption or chemisorption) of azodyes. In one of our previous studies, DFT studies have been used for the comprehensive understanding of energetics and electron transfer mechanism of silver capped rGO for the photocatalytic degradation of EBT and MO. In this work, DFT simulations revealed that nature of adsorption of water contaminant azo dyes on the surface of GO was physiosorption (Fig 7) as evident from top-view and side-view of dyes on the GO surface. Using an advanced DFT approach the adsorption energy was calculated with excellent accuracy.

**Fig 8:**
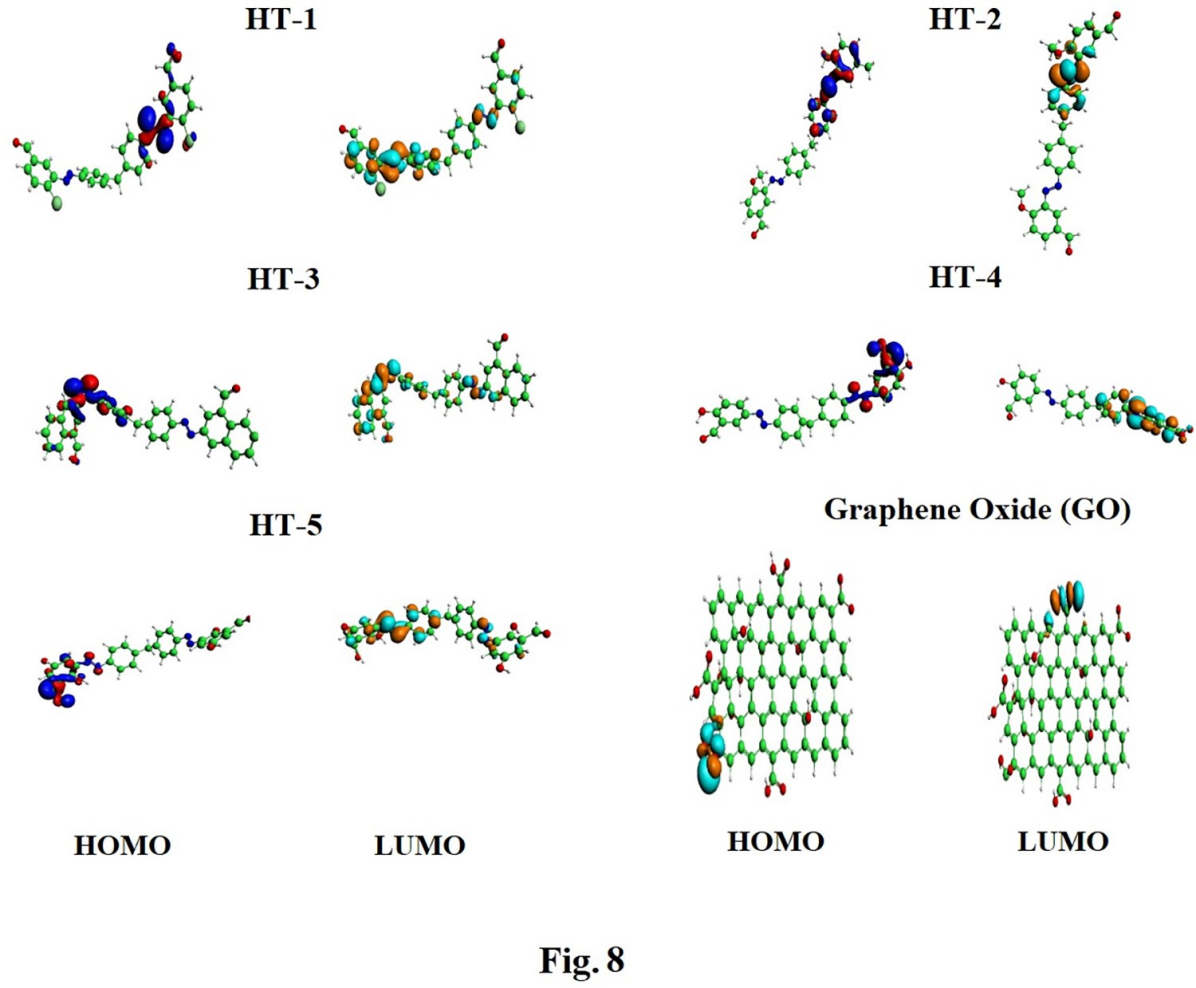
Frontier molecular orbital analysis of azo dyes adsorbed showing distribution of isodensities indicating their reactive parts of azo dyes.

**Fig 9:**
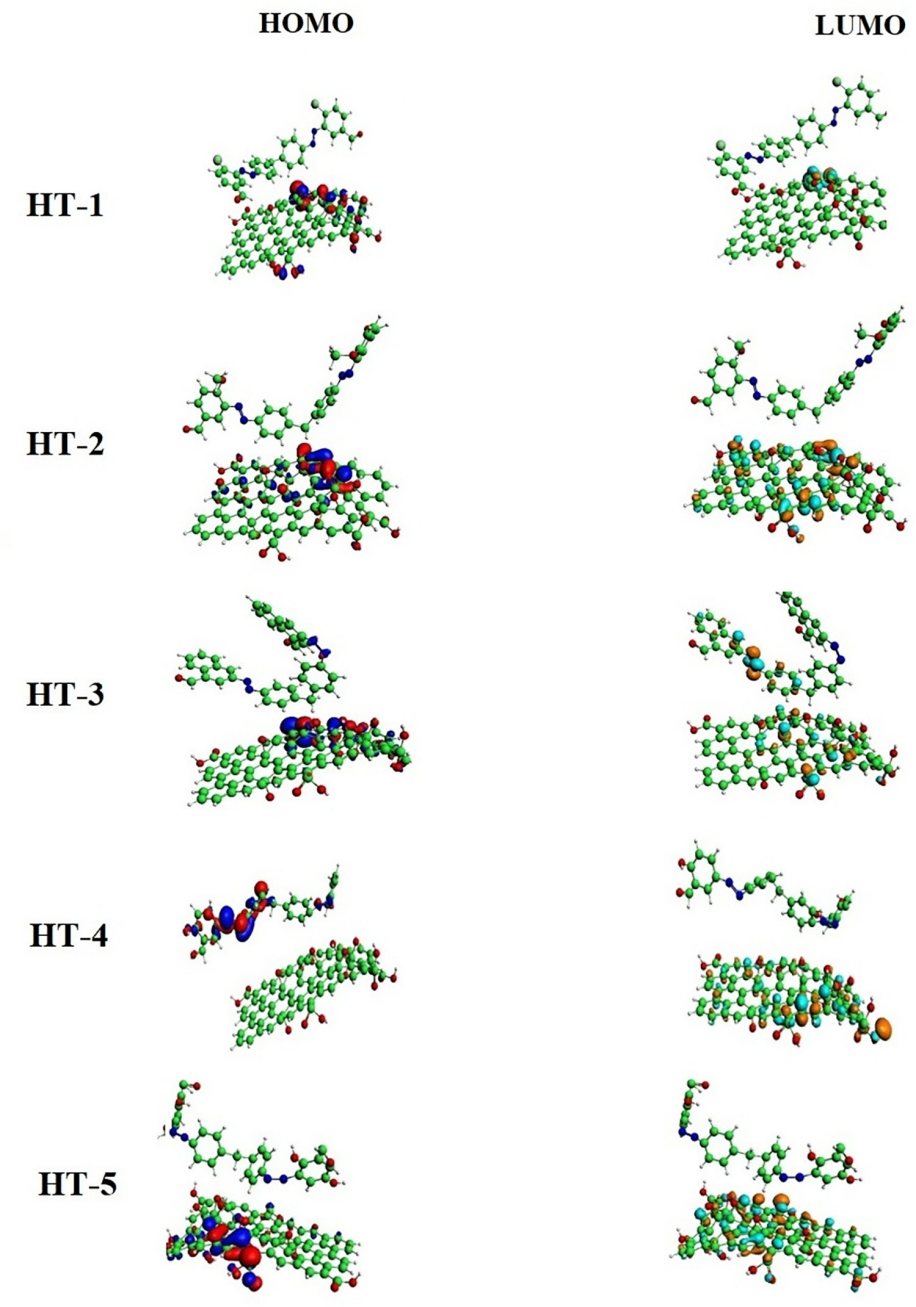
Frontier molecular orbital analysis of azo dyes adsorbed on GO surface showing distribution of HOMO and LUMO isodensities on GO and azo dyes.

Energies of frontier molecular orbitals *i*.*e*., E_HOMO_ and E_LUMO_ along with their isodensities distributions were calculated to monitor electrophilic or nucleophilic character of GO surface while acting as adsorbent. As evident from the Figs 8 and 9 that most distribution of HOMO isodensities are located on–C = O part of GO reflecting its electron donating behavior while interacting with HT-1, HT-2, HT-3 and HT-5. However, for HT-4, HOMO isodensities are distributed over HT-4 instead of GO, whereas LUMO isodensities are spread over all the GO surface indicative of strong electron accepting behavior of GO in the presence of HT-4 as adsorbate. For comprehensive understanding of electron transfer feasibility from E_HOMO_ to E_LUMO_, ΔE (E_HOMO_-E_LUMO_ gap) was determined as depicted in Table 1.

**Table 1:** Adsorption distances, frontier orbital energies (E_HOMO_, E_LUMO_) and ΔE = E_HOMO_-E_LUMO_ showing electronic interactions after adsorption.

Adsorption	Distance (Å)	E_HOMO_ kcal/mol	E_LUMO_ kcal/mol	ΔE = E_HOMO_-E_LUMO_ kcal/mol
(HT-1)+GO	3.5	-134.45	-133.67	0.77
(HT-2)+GO	3.5(317–318)	-74.73	-72.91	1.82
(HT-3)+GO	3.5(313–314)	-111.61	-103.65	7.95
(HT-4)+GO	3.5(324–325)	-53.59	-51.96	1.63
(HT-5)+GO	3.5(314–315)	-82.60	-81.54	1.06

Distribution of isodensities of azodyes were determined to assess their comparative reactivity. Smaller HOMO-LUMO gap implies feasible electrons transfer from E_HOMO_ to E_LUMO_ and hence maximum greater possibility of the interactions as revealed by Table 1. It was observed that HOMO-LUMO gap for azo dyes HT-1, HT-2, HT -4 and HT 1–5 was significantly smaller indicating greater electronic overlapping at distance of 3.5 Å while azo dye HT-3 showed comparatively larger HOMO-LUMO gap was high *i*.*e*., -7.95 kcal/mol hence lesser reactivity as because of its stable 1-naphthoaldehyde coupler.

To probe into the fact that adsorption is thermodynamically favorable or not, thermodynamic parameters (*ΔG*, *ΔH* and *ΔS)* were calculated based on frequency calculation using GGA: PBE and electron correlation approximations. Table 2 tabulates thermodynamic parameters for five azodyes adsorbed at the distance of 3.5 Å on the surface of GO. A negative value of *ΔG* and *ΔH* represented that adsorption of all dyes on GO surface is thermodynamically favored and feasible. To find out strength of molecular interaction with azodyes and GO surface, adsorption constant K_ad_ was evaluated and indicated in Table 2. Computational data revealed that K_ad_ values for HT-4 was found to be highest due to its coupler. *i*.*e*. 2-hydroxybenzaldehyde. Coupler formed strong intermolecular interaction between its -OH, -CHO with–COC- group of GO. These trend are consistent with experimental results which showed highest value of K_L_ for HT-4. On the other hand, HT-3 furnished lowest affinity of adsorption on the surface of GO as compared to other azo dyes, because of very stable, conjugated and aromatic 1-naphthaldehyde coupler. Moreover, *ΔG* also revealed that azo dye HT-4 has greater spontaneity and HT-3 has least spontaneity as evident in Table 3.

**Table 2:** Comparison of Gibb’s free energy (ΔG) and adsorption constant calculated from experimental and computational results.

Azo dye adsorption	Computational DFT data			Experimental data	
	K_ad/_(M^-1^)	Gibbs Free Energy (ΔG) kJ/mol		K_L_(M^-1^)	Gibbs Free Energy (ΔG) kJ/mol
(HT-1) + GO	1.13×10^10^		-57.37	1.94×10^9^	-52.99
(HT-2) + GO	1.53×10^9^		-52.41	1.37×10^9^	-52.13
(HT-3) + GO	2.42×10^5^		-30.72	7.08×10^8^	-50.49
(HT-4) + GO	1.93×10^10^		-58.69	3.76×10^9^	-54.62
(HT-5) + GO	8.74×10^8^		-51.01	1.55×10^9^	-52.43

**Table 3:** Kinetics and thermodynamic parameter of adsorption at 3.5Å distance between azo dyes (HT 1–1), (HT 1–2), (HT 1–3), (HT 1–4), (HT 1–5) and graphene oxide at temperature 298K calculated using DFT.

Adsorption	Enthalpy	Entropy	Heat Capacity	Gibbs Free Energy	K_ad_
	*ΔH (*kJ/mol)	*ΔS* (kJ/mol-K)	*C*_*v*_ (kJ/K)	*ΔG* (kJ/mol)	(M^-1^)
(HT-1)+GO	182.13	0.83	0.61	-57.37	1.13×10^10^
(HT-2)+GO	164.7	0.79	0.55	-52.41	1.51×10^9^
(HT-3)+GO	55.73	0.5	0.184	-30.72	2.42×10^5^
(HT-4)+GO	187.02	0.83	0.64	-58.69	1.93×10^10^
(HT-5)+GO	125.89	0.66	0.42	-51.01	8.74×10^8^

**Fig 10:**
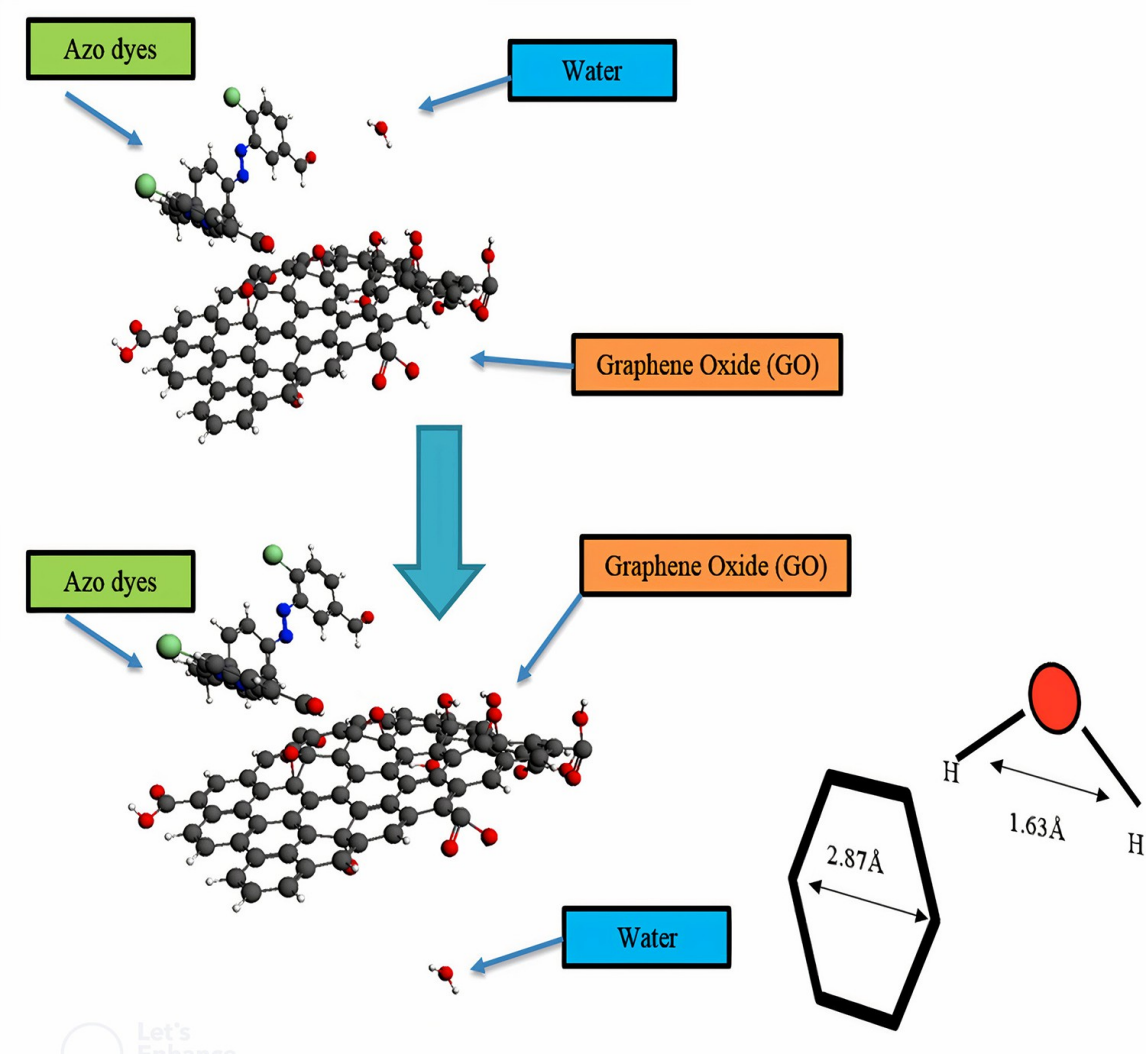
Passage of water molecules through graphene hexagon leaving behind large sized azo dyes; Graphene is acting as molecular sieve.

The movement of water molecules and azo dyes from the GO molecular sieves hexagonal structure was determined by frequency calculations. The result revealed that water can easily passed through hexagon of GO due to small size of water molecule which is 1.63Å width while GO hexagon has 2.87Å. On the other hand, azo dyes are large sized 19.18 Å approximately and unable to pass through 2.78 Å hexagon as shown in Fig 10.

Interaction of azo-dyes and GO resulted in Vander Waal’s interactions with no chemical bond formation, thus demonstrating that azo dyes adsorbed on the surface of GO *via* physiosorption. Due to physiosorption of azo dyes on GO, it can easily be recycled through desorption mechanism. Both experimental and computational results revealed that the azo-dye namely Bis 3,3’-(4,4’-Diazenyldiphenylmethane)-naphthaldehyde (HT-3) had lowest adsorption constant values due to intramolecular π-π interaction between its coupler rings; while the azo-dye namely, Bis 3,3’-(4,4’-Diazenyldiphenylmethane)-salicylaldehyde (HT-4) had highest adsorption constant values due to–OH and–COC-group of GO.

#### 3.7.2 Adsorption through Monte Carlo simulations

**Fig 11:**
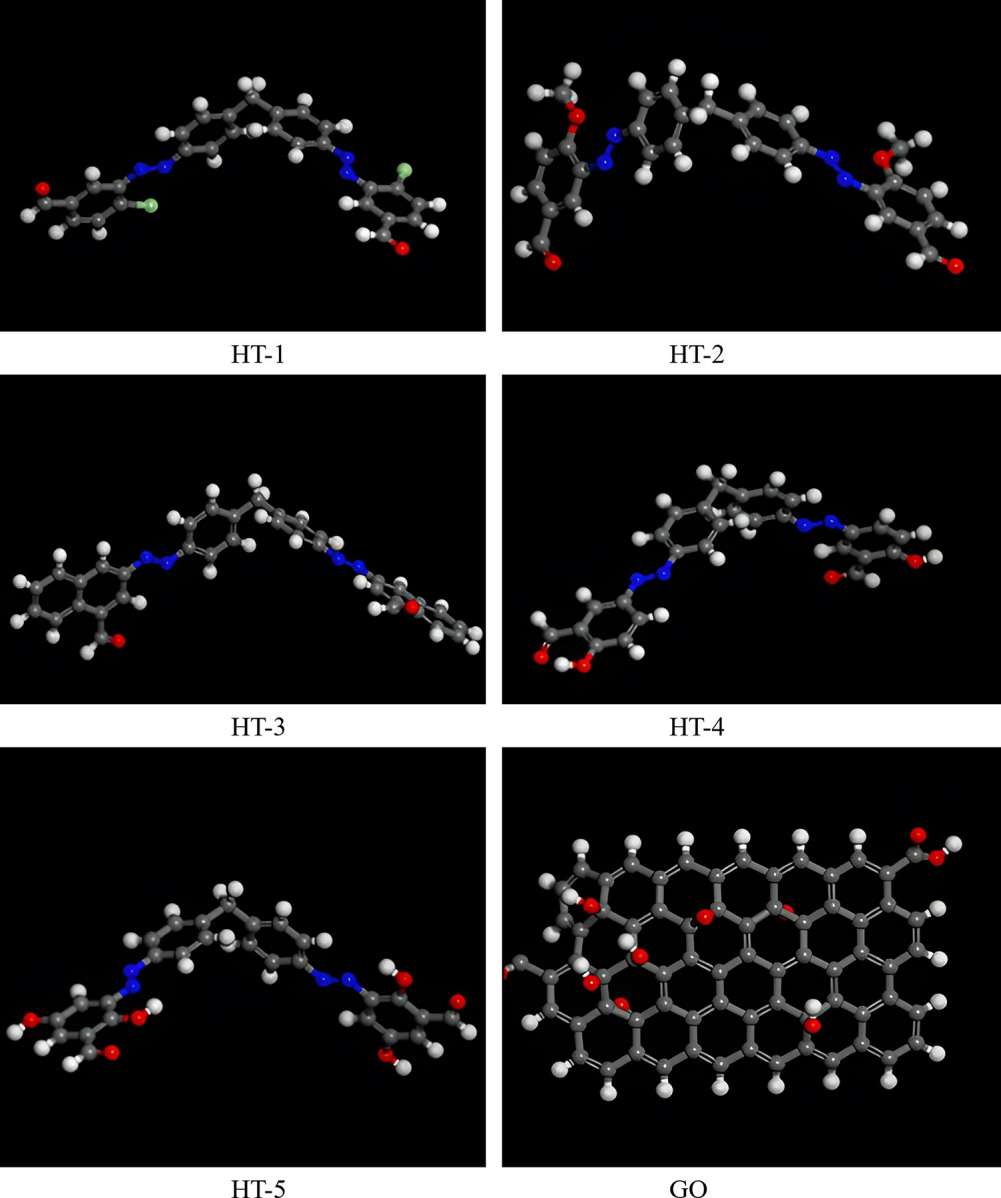
Optimized structures of organic dyes and GO through FORCITE module of Material Studio.

**Fig 12:**
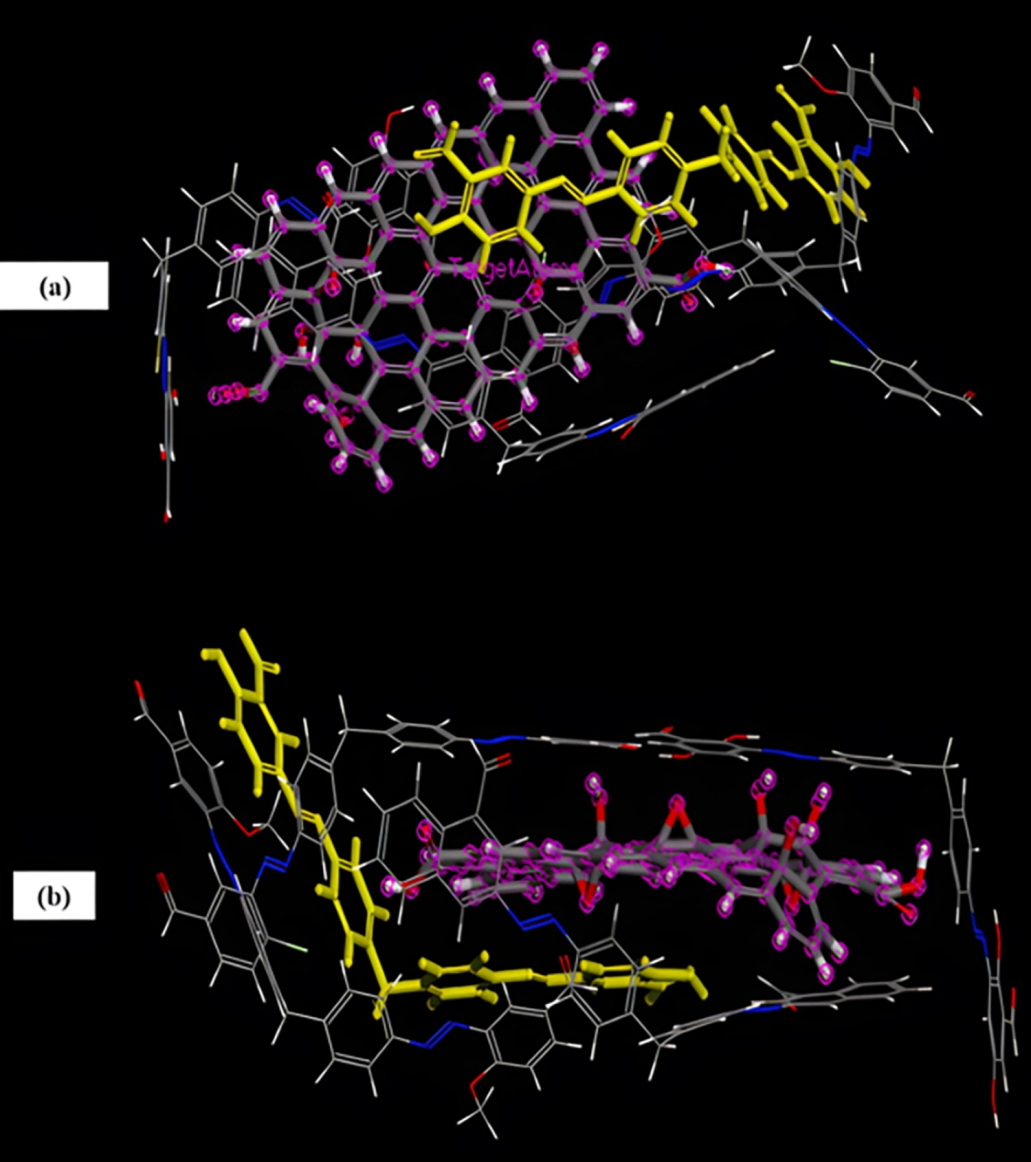
Adsorption complex of organic dyes and GO (HT-4 is shown in yellow color; a and b are top and side views, respectively).

The optimized structures of dyes and GO are depicted in Fig 11 and the corresponding calculated energies are presented in Table 4. The total energy is the sum of adsorption energy, rigid adsorption energy, and deformation energy. Adsorption energy is the amount of energy released or emitted when relaxed dye adsorbed on GO, whereas rigid adsorption energy is released by adsorption of unrelaxed dyes on the GO surface. Other type of energy, i.e., deformation energy is released when adsorbed dyes are relaxed on GO surface. The energy required to separate a dye from the surface of graphene oxide is represented by DE_ad_/DN_i_. The obtained lowest energy configuration for dyes adsorption on GO is depicted in Fig 12. The obtained adsorption complex revealed that GO provides active sites and functional groups which enhanced the feasibility and possibility of adsorption of dyes. However, the favourable adsorption site for dyes on GO is the benzene ring and hydroxyl group. The observed interactions between GO and dye’s complexes are found to be intermolecular hydrogen bonding and π-π interactions. Tabular values of DE_ad_/dNi shows that greater amount of energy i.3., -67.5352 Kcal/mol is required to separate HT-4 from GO as compared to other dyes because of its stronger adsorption with adsorbent surface thereby indicating greater effectiveness of GO in removal of HT-4.

**Table 4:** Calculated energies of adsorption complex of dyes and GO.

**Total Energy** (Kcal/mol)	185.0328
**Adsorption Energy** (Kcal/mol)	-178.1723
**Rigid Adsorption Energy**(Kcal/mol)	-184.5230
**Deformation Energy**(Kcal/mol)	6.3507
**HT-1**	-45.6128
**dE**_**ad**_**/dN**_**i**_ (Kcal/mol)	
**HT-2**	-44.2370
**dE**_**ad**_**/dN**_**i**_ (Kcal/mol)	
**HT-3**	-51.7192
**dE**_**ad**_**/dN**_**i**_ (Kcal/mol)	
**HT-4**	-67.5352
**dE**_**ad**_**/dN**_**i**_ (Kcal/mol)	
**HT-5**	-34.7123
**dE**_**ad**_**/dN**_**i**_ (Kcal/mol)	

## 4. Conclusions

Herein, we explored the adsorption of five novel azodyes onto the surface of GO through a combination of experimental UV-Vis spectroscopic method and computational DFT and Monte Carlo methods. We systematically investigated the adsorption of azo-dyes on GO, considering adsorbent dosage, contact time, and the Langmuir adsorption isotherm. The experimental data were explained by the Langmuir isotherm model, and the adsorption kinetics was modeled successfully by pseudo-second-order rate equation.

Computational DFT and Monte Carlo results revealed a predominantly physisorption nature in the adsorption process of azo-dyes on GO. The negatively charged surface, decorated with oxide functional groups, exhibited higher adsorption of HT-4 compared to other azodyes. Non-covalent and charge interactions were identified as driving forces for the adsorption on GO. Electronic and thermodynamic parameters calculated through DFT and MC simulation confirmed favorable adsorption of all azodyes, HT-4 being most efficient to be adsorbed on GO surface as revealed by its lowest ΔE_ads_. Both experimental and computational findings, particularly thermodynamic parameters, were mutually supportive. The physisorption depicted reusability and potential of GO as an effective adsorbent for removing these novel azo dyes from aqueous solutions. Our results contribute to the development of efficient adsorbents for the broader removal of dyes in water purification applications.

## Supporting information

S1 Fig **(a-e)** FT-IR, XRD, TGA, SEM, and UV-spectra, respectively, of GO. **(f)** FTIR of recycled GO.
(TIF)

S2 Fig IR and NMR spectra of HT-2. (TIF)

S1 Table Supporting information data for pseudo-first-order and pseudo-second-order kinetic modeling of azo-dyes adsorption on GO. (XLSX)

S1 Scheme Synthesis of substituted Bis 3, 3’-(4, 4’-diazenyldiphenylmethane) benzaldehydes. (PNG)

S1 Graphical abstract (TIF)
