# Supplementary figures and images for: Efficient removal of toxic azo dyes from contaminated water by adsorption on the GO surface
Source: PLoS One. 2024 Mar 29;19(3):e0299364. doi: 10.1371/journal.pone.0299364 (PMC10980192; doi:10.1371/journal.pone.0299364)

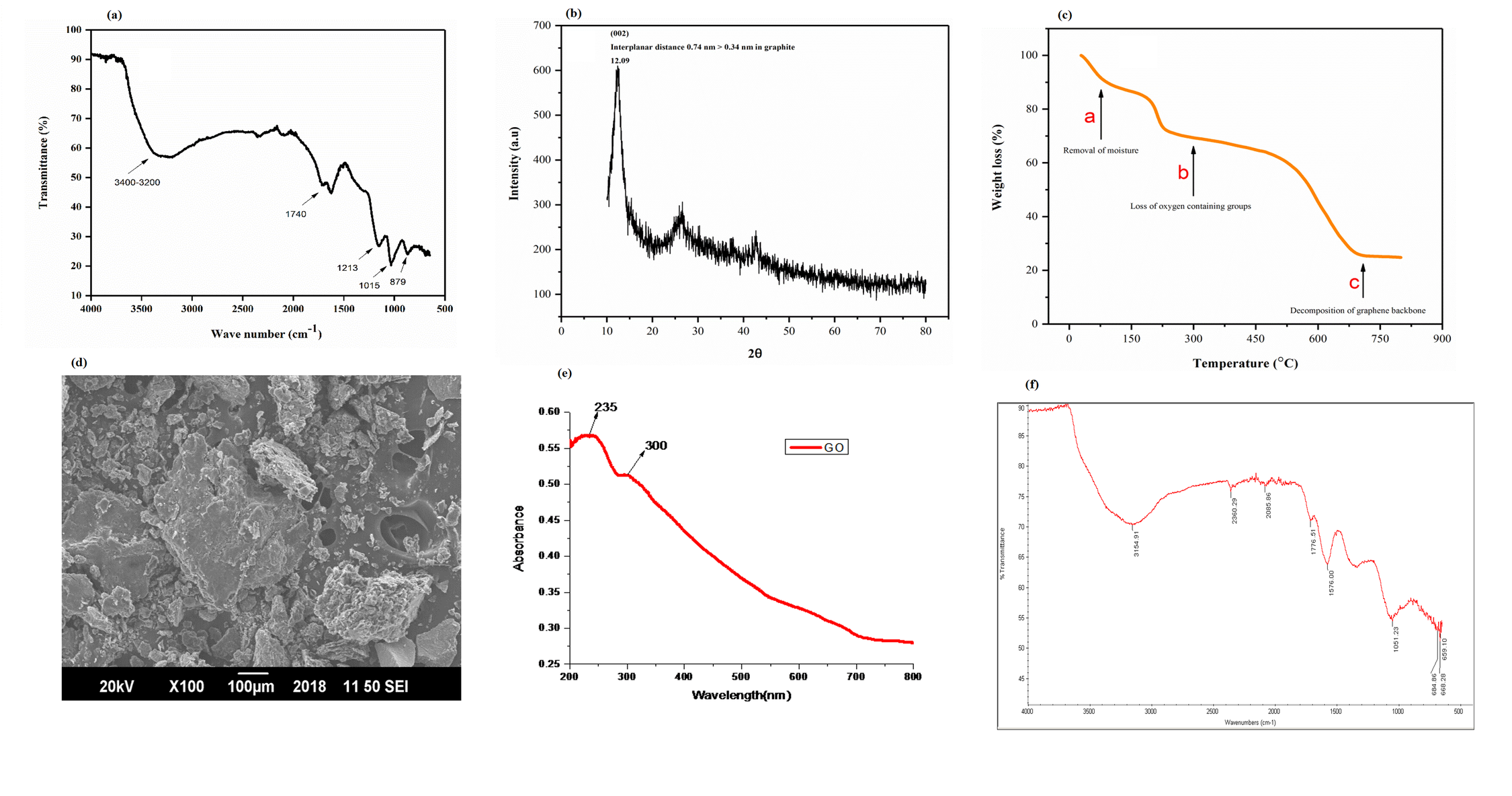

Supplement: S1 Fig — (a-e) FT-IR, XRD, TGA, SEM, and UV-spectra, respectively, of GO. (f) FTIR of recycled GO. (TIF) [file pone.0299364.s001.tif]

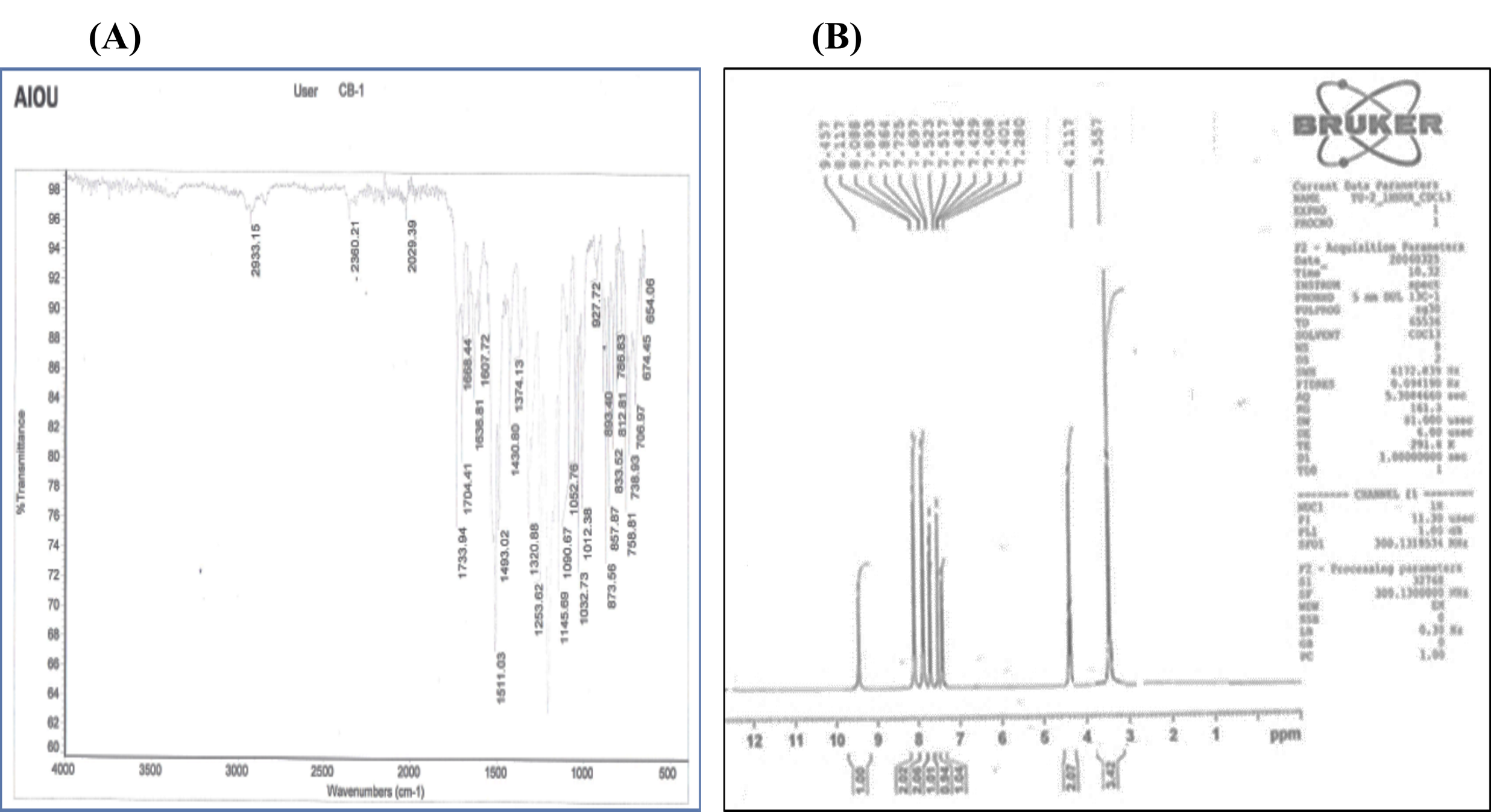

Supplement: S2 Fig — (TIF) [file pone.0299364.s002.tif]

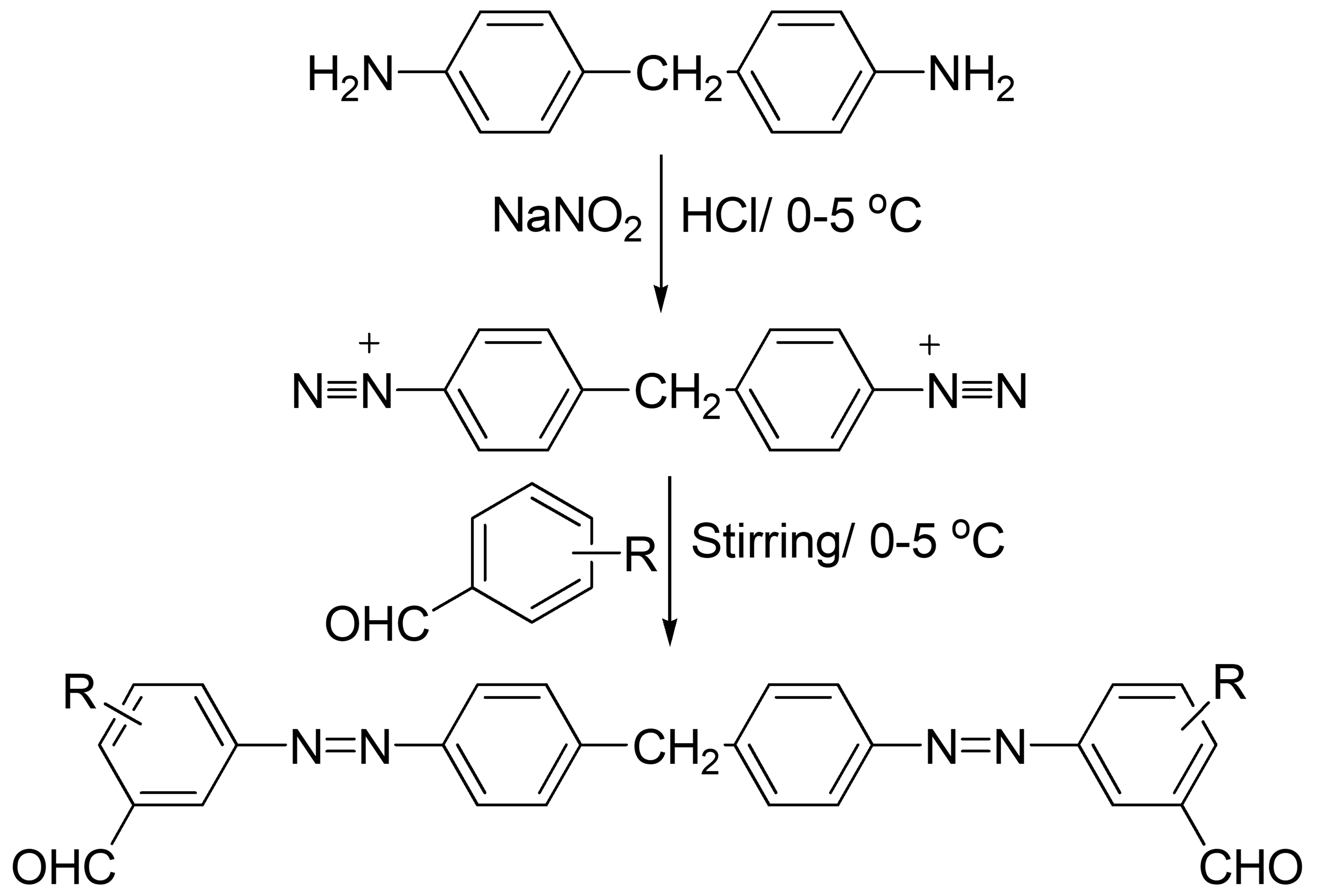

Supplement: S1 Scheme — (PNG) [file pone.0299364.s004.png]

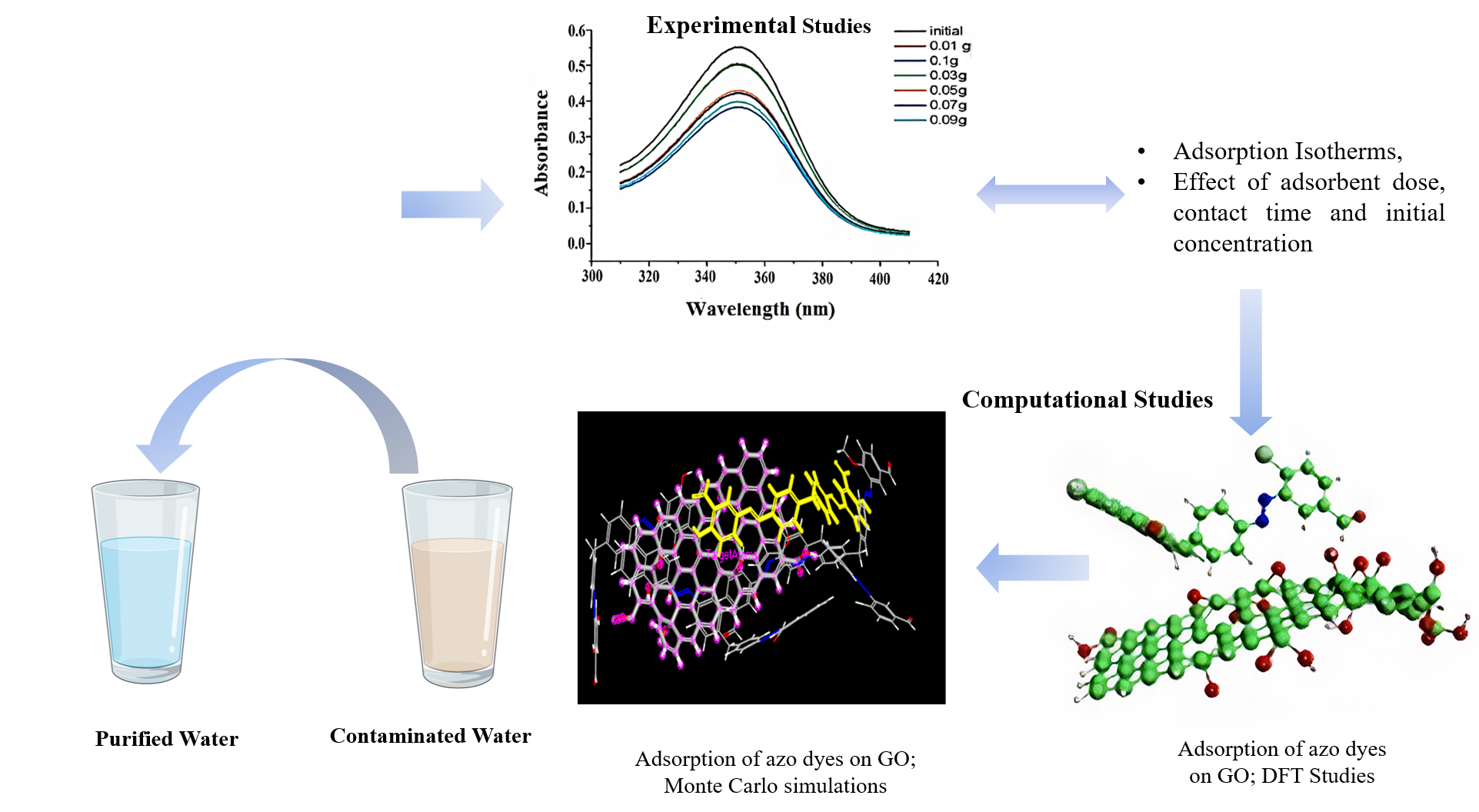

Supplement: S1 Graphical abstract — (TIF) [file pone.0299364.s005.tif]
